# Severe dyslipidemia and concomitant risk factors in the middle-aged Lithuanian adults: a cross-sectional cohort study

**DOI:** 10.1186/s12944-018-0731-7

**Published:** 2018-04-19

**Authors:** Sandra Kutkienė, Žaneta Petrulionienė, Aleksandras Laucevičius, Marija Petrylaitė, Diana Maskeliūnaitė, Roma Puronaitė, Milda Kovaitė, Irma Kalibaitaitė, Egidija Rinkūnienė, Vilma Dženkevičiūtė, Vytautas Kasiulevičius

**Affiliations:** 1 Vilnius University, Faculty of Medicine, Vilnius, Lithuania; 2 Vilnius University, Faculty of Medicine, Clinic of Cardiac and Vascular Diseases, Vilnius, Lithuania; 3 Vilnius University, Faculty of Medicine, Clinic of Internal Diseases, Family Medicine and Oncology, Vilnius, Lithuania; 4 Vilnius University Hospital Santaros Klinikos, Vilnius, Lithuania

**Keywords:** Severe dyslipidemia, Cardiovascular risk factors, Primary prevention

## Abstract

**Background:**

Dyslipidemia is highly prevalent and is one of the major risk factors for cardiovascular disease in Lithuania. The purpose of this study was to determine the prevalence of severe dyslipidemia in Lithuanian middle aged primary prevention population and to investigate cardiovascular risk profile.

**Methods:**

The group of 83,376 people were examined in the Lithuanian High Cardiovascular Risk primary prevention program (LitHiR), during 2009–2015 years. This study recruited middle aged men and women without overt cardiovascular disease. The prevalence of cardiovascular risk factors was compared between severe dyslipidemia group and *control group.*

**Results:**

Severe dyslipidemia was present in 13.5% (11265) of the subjects; 66.6% (7508) were females. The subjects with severe dyslipidemia had significantly higher rates of arterial hypertension (63.5% vs. 44.2%, *p* < 0.001), diabetes mellitus (16% vs. 8.1%, *p <* 0,001), abdominal obesity (51% vs. 30.3%, *p* < 0.001), body mass index (BMI) > 30 (kg/m^2^) (38.8% vs. 24.1%, *p* < 0.001), metabolic syndrome (47.2% vs. 9.2%, *p* < 0.001), unbalanced diet (66.5% vs. 53.5%, *p* < 0.001), insufficient physical activity (56% vs. 44.2%, *p* < 0.001), family history of cardiovascular disease (29.7% vs. 22.7%, *p* < 0.001) in comparison with control group. Subjects without dyslipidemia had significantly higher rates of smoking (26.4% vs. 22.7%, *p* < 0.001).

The prevalence of familial hypercholesterolemia was 0.1%, very high hypertriglyceridemia - 0.2% and familial mixed dyslipidemia - 0.1% of the subjects examined in the LitHiR programme.

****Conclusions**:**

High prevalence of dyslipidemia remains a major problem in Lithuania. 9 out of 10 people have dyslipidemia, 1 out of 10 - severe dyslipidemia. Severe dyslipidemia is associated with higher frequency of other cardiovascular risk factors.

## Background

Although mortality rates from cardiovascular diseases have shown a remarkable decline in many Western countries, cardiovascular mortality in Lithuania has remained high [1]. Several studies on prevalence of cardiovascular risk factors have been conducted in Lithuania. CINDI survey in 2007 proved that hypercholesterolemia was identified in every second examined person without gender differences [2]. Another MONICA study showed that prevalence of hypercholesterolemia remained unchanged and very high in both men (80.7%) and women (82.7%) in 1983–2002 [3]. However, they contained some limitations, such as the following: the analysis only of certain geographic regions as in MONICA study only citizens of Kaunas city were examined [3] and in CINDI project citizens of only five Lithuania regional centers were checked [2]; the number of analyzed subjects was also quite low as well. In reference to the unfavorable situation with cardiovascular morbidity and mortality in Lithuania, the Lithuanian High Cardiovascular Risk programme (LitHiR) started in 2006. The aim of this project was to develop a clinically oriented nationwide programme for the assessment of cardiovascular risk in subjects of employable age without overt CVD, with a view to pursuing aggressive modification of risk factors in those with elevated risk. First results of this programme showed a very high prevalence of dyslipidemia (89.7%) [4]. 91.3% of these patients had an increased rate of total cholesterol (TC) and 88.6% had an increased rate of low density lipoprotein – cholesterol (LDL-C) [5]. Unfortunately, severe dyslipidemia group of this population has never been analyzed before.

The aim of this study was to determine the prevalence of severe dyslipidemia in Lithuanian middle aged primary prevention population, to investigate cardiovascular risk profile of these patients and to compare them with people of the *control group.*

## Methods

The Lithuanian High Cardiovascular Risk (LitHiR) Primary Prevention Programme for the identification of patients at high-risk of cardiovascular disease and the implementation of the methods of primary prevention (hereinafter referred to as LitHiR programme) was launched in Lithuania in 2006. The study recruited 40–55 years old men and 50–65 years old women without overt cardiovascular disease (CVD) from all Lithuanian regions. Over the period of 2009 to 2015, the total of 83.376 persons were analyzed. The severe dyslipidemia group, which was in detail analyzed, consist of 11.265 persons. Severe dyslipidemia was considered if TC ≥ 7.5 mmol/l, or LDL-C ≥ 6 mmol/l, or triglycerides (TG) ≥ 4.5 mmol/l. High density lipoprotein cholesterol (HDL-C) was not involved, because of controversial discussions about HDL-C clinical importance and benefit of absolute quantity of HDL-C. Participants underwent risk profile – lifestyle (smoking, physical activity, dietary patterns) – analysis, personal and family patterns for CVD in the first degree blood relatives, anthropometry (height, weight, waist circumference and body mass index (BMI), defined as weight in kilograms divided by height (in square meters), blood pressure and pulse determination). Blood pressure was measured in a sitting position after at least a 5-min rest in a dominant arm. The participant’s dominant arm was supported at heart level and correctly sized cuffs were used. It was recommended to take the average of three measurements. Serum TC, HDL-C, TG, calculated LDL-C was carried out and plasma glucose was sampled for the estimation of fasting blood glucose levels. The overall cardiovascular risk according to the risk estimation SCORE (Systematic Coronary Risk Evaluation) system was calculated.

Patients without dyslipidemia were included in control group if TC ≤ 5 mmol/l, LDL-C ≤ 3 mmol/l, TG ≤1.7 mmol/l and HDL-C > 1.0 mmol/l in men and > 1.2 mmol/l in women.

All patients of severe dyslipidemia group were divided in 3 subgroups according blood lipids levels [5]: severe hypercholesterolemia group, which consisted of 3 smaller subgroups: group of high LDL-C (≥ 6 mmol/l), possible familial hypercholesterolemia (FH) group (LDL-C = 6.5–8.4 mmol/l + family history of CVD) and definite FH group (LDL-C ≥ 8.5 mmol/l) (12); severe hypertriglyceridemia group, which consisted of 2 subgroups: group with high level of TG (≥ 4.5 mmol/l) and group with very high TG elevation (≥ 10 mmol/l) (categorization was suggested by local LitHiR steering committee after assessing situation in Lithuania) and mixed dyslipidemia group (TG ≥ 4.5 mmol/l and LDL-C ≥ 6 mmol/l in same person) (criteria were suggested by local LitHiR steering committee after assessing situation in Lithuania).

Arterial hypertension was determined when arterial blood pressure was ≥140 mmHg and/or diastolic blood pressure was ≥90 mmHg, or the diagnosis of treated hypertension was documented in a medical record. Metabolic syndrome (MS) was assessed according to the National Cholesterol Education Program III modified criteria [6]. An unbalanced diet was defined as eating food not having enough fiber, exceeding 30% of the fat percentage of calorie count, having total fat count composed of more than one third of saturated fat or consuming more than 300 mg of cholesterol per day. Insufficient physical activity was considered as doing aerobic exercises less than three times a week with sessions shorter than half an hour.

The calculation of the total number of risk factors was based on the following binary risk factors: hypertension, dyslipidemia, diabetes mellitus (DM), abdominal obesity, smoking, MS, inadequate physical activity, unbalanced diet and family history of CVD.

### Statistical analysis

For continuous variables the following descriptive statistics are reported: means, standard deviations (SD), 95% confidence interval (CI). For categorical data frequencies are reported. In case of dichotomous categorical variables we also provide confidence intervals for proportions of interest (e.g., diabetes, smoking, etc.). These intervals are obtained using the relationship between beta and binomial distributions.

Continuous variables were compared with a help of the t-test or the Mann-Whitney test. Categorical variables were compared with a help of the chi-square test. All reported *p*-values are two-tailed. The level of significance was set to 0.05. The SPSS version 22 (SPSS Inc., Chicago, Illinois, USA) statistical package was used for all statistical calculations.

## Results

### Baseline characteristics

A total of 83.376 patients were analyzed, during the years of 2009–2015. The mean age was 52.3 ± 6.18 years, 59.1% (49234) were females. The prevalence of severe dyslipidemia in Lithuania was 13.5% (11265); 66.6% (7508) were females.

The risk profile of severe dyslipidemia group and control group were analyzed in Table 1.

**Table 1:** Cardiovascular risk profile of severe dyslipidemia and control group

	Control group (*n* = 8.538)	Severe dyslipidemia (*n* = 11.265)	*p* value
	Average ± SD (95% CI)	Average ± SD (95% CI)	
Age (years)	50.7 ± 6.04 (50.57–50.83)	53.51 ± 6.21 (53.4–53.63)	< 0.001
Waist circumference (cm)	90.64 ± 13.11 (90.36–90.92)	94.68 ± 13.02 (94.44–94.92)	< 0.001
BMI (kg/m^2^)	27.08 ± 5.43 (26.96–27.19)	29.11 ± 5.01 (29.02–29.2)	< 0.001
SBP (mmHg)	130.58 ± 15.43 (130.26–130.91)	136.36 ± 17.21 (136.04–136.68)	< 0.001
DBP (mmHg)	81.19 ± 9.12 (80.99–81.38)	84.19 ± 9.78 (84.01–84.37)	< 0.001
Heart rate (beats/min)	71.52 ± 8.7 (71.33–71.7)	72.59 ± 8.68 (72.43–72.75)	< 0.001
Glucose (mmol/l)	5.34 ± 1.07 (5.32–5.37)	5.74 ± 1.57 (5.71–5.77)	< 0.001
TC (mmol/l)	4.41 ± 0.44 (4.4–4.42)	8.08 ± 0.99 (8.06–8.09)	< 0.001
LDL-C (mmol/l)	2.42 ± 0.43 (2.41–2.43)	5.35 ± 1.19 (5.33–5.37)	< 0.001
HDL-C (mmol/l)	1.58 ± 0.36 (1.57–1.59)	1.6 ± 0.51 (1.59–1.61)	0.005
TG (mmol/l)	0.93 ± 0.31 (0.93–0.94)	2.65 ± 2.27 (2.61–2.7)	< 0.001
SCORE index (points)	1.22 ± 1.19 (1.2–1.25)	2.79 ± 2.26 (2.75–2.83)	< 0.001
Arterial Hypertension (%)	44.2 (43.1–45.3)	63.5 (62.6–64.4)	< 0.001
Diabetes mellitus (%)	8.1 (7.5–8.7)	16 (15.3–16.7)	< 0.001
Abdominal obesity (%)	30.3 (29.3–31.3)	51 (50.1–51.9)	< 0.001
BMI > 30 (kg/m^2^) (%)	24.1 (23.2–25)	38.8 (37.9–39.7)	< 0.001
Smoking (%)	26.4 (25.5–27.4)	22.7 (21.9–23.5)	< 0.001
Metabolic syndrome (%)	9.2 (8.6–9.9)	47.2 (46.3–48.1)	< 0.001
RF ≥ 3 (%)	44.1 (43.1–45.2)	84.5 (83.8–85.1)	< 0.001
Family history of CVD (%)	22.7 (21.8–23.6)	29.7 (28.9–30.6)	< 0.001
Diet (unbalanced) (%)	53.5 (52.4–54.5)	66.5 (65.6–67.4)	< 0.001
Physical activity (insufficient) (%)	44.2 (43.2–45.3)	56 (55.1–56.9)	< 0.001

*Abbreviations*: *BMI* body mass index, *SBP* systolic blood pressure, *DBP* diastolic blood pressure, *TC* total cholesterol, *LDL–C* low-density lipoprotein cholesterol, *HDL-C* high-density lipoprotein cholesterol, *TG* triglycerides, *BMI* body mass index, *RF* risk factors, *CVD* cardiovascular disease, *SCORE index* Systematic Coronary Risk Evaluation index

This study found, that people with severe dyslipidemia are two times more likely to have three or more risk factors than people in control group. The subjects with severe dyslipidemia had significantly higher rates of arterial hypertension, abdominal obesity, body mass index (BMI) > 30 (kg/m^2^), diabetes mellitus, unbalanced diet, insufficient physical activity, family history of CVD. Metabolic syndrome was approximately 5 times more prevalent in patients with severe dyslipidemia than in control group (Table 1).

### Gender differences in risk profile

A total of 7.508 females and 3.575 males with severe dyslipidemia were analyzed. The mean age of females was 56.96 ± 4.29 years; and 47.17 ± 4.28 years, of males, *p* < 0.001.

The risk profile of males and females, who have established severe dyslipidemia were analyzed. (Table 2).

**Table 2:** Cardiovascular risk profile of men and women with severe dyslipidemia

	Female (*n* = 7.508)	Male (*n* = 3.757)	*p* value
	Average ± SD (95% CI)	Average ± SD (95% CI)	
Age (years)	56.69 ± 4.29 (56.59–56.78)	47.17 ± 4.28 (47.03–47.3)	< 0.001
Waist circumference (cm)	91.97 ± 12.75 (91.68–92.25)	100.1 ± 11.81 (99.73–100.48)	< 0.001
BMI (kg/m^2^)	29.08 ± 5.21 (28.97–29.2)	29.16 ± 4.57 (29.02–29.31)	0.404
SBP (mmHg)	136.22 ± 17.42 (135.83–136.62)	136.63 ± 16.79 (136.09–137.17)	0.233
DBP (mmHg)	83.54 ± 9.49 (83.33–83.76)	85.49 ± 10.21 (85.17–85.82)	< 0.001
Heart rate (beats/min)	72.41 ± 8.31 (72.23–72.6)	72.94 ± 9.35 (72.64–73.24)	0.004
Glucose (mmol/l)	5.63 ± 1.39 (5.6–5.66)	5.96 ± 1.86 (5.9–6.02)	< 0.001
TC (mmol/l)	8.2 ± 0.86 (8.18–8.22)	7.83 ± 1.16 (7.8–7.87)	< 0.001
LDL-C (mmol/l)	5.53 ± 1 (5.5–5.55)	5 ± 1.45 (4.96–5.05)	< 0.001
HDL-C (mmol/l)	1.71 ± 0.48 (1.7–1.72)	1.38 ± 0.49 (1.36–1.39)	< 0.001
TG (mmol/l)	2.17 ± 1.64 (2.13–2.2)	3.63 ± 2.95 (3.53–3.72)	< 0.001
SCORE index (points)	2.75 ± 2.17 (2.71–2.8)	2.85 ± 2.41 (2.78–2.93)	0.036
Arterial Hypertension (%)	65.4 (64.3–66.4)	59.7 (58.1–61.3)	< 0.001
Diabetes mellitus (%)	14.7 (13.9–15.5)	18.6 (17.3–19.8)	< 0.001
Abdominal obesity (%)	57.2 (56–58.3)	38.6 (37.1–40.2)	< 0.001
BMI > 30 (kg/m^2^) (%)	38.8 (37.7–40)	38.6 (37.1–40.2)	0.823
Smoking (%)	12.4 (11.6–13.1)	43.3 (41.7–44.9)	< 0.001
Metabolic syndrome (%)	46.6 (45.5–47.8)	48.4 (46.8–50)	0.071
RF ≥ 3 (%)	83.6 (82.7–84.4)	86.2 (85–87.3)	< 0.001
Family history of CVD (%)	31.2 (30.1–32.2)	26.8 (25.4–28.3)	< 0.001
Diet (unbalanced) (%)	63.4 (62.3–64.5)	72.7 (71.3–74.1)	< 0.001
Physical activity (insufficient) (%)	57.2 (56–58.3)	53.7 (52.1–55.3)	< 0.001

*Abbreviations*: *BMI* body mass index, *SBP* systolic blood pressure, *DBP* diastolic blood pressure, *TC* total cholesterol, *LDL-C* low-density lipoprotein cholesterol, *HDL-C* high-density lipoprotein cholesterol, *TG* triglycerides, *RF* risk factors, *CVD* cardiovascular disease, *SCORE index* Systematic Coronary Risk Evaluation index

A significant difference in the prevalence of arterial hypertension, abdominal obesity, family history of CVD and insufficient physical activity between females and males was found – females more often had arterial hypertension, were more obese, they more often had positive family history of CVD and were less active. Males significantly more often had diabetes mellitus and they also had significantly higher SCORE index in comparison with females. Furthermore, a considerable difference in the prevalence of smoking between males and females was found - the number of smoking males was four times higher than the number of smoking females (Table 2).

### The prevalence of familial lipid disorders

The total number of the participants in the analysis was 83.376. High LDL-C was present in 3.4% (2827) of the subjects. Possible FH – 0.5% (389) and definite FH - 0.1% (65) was defined of the subjects. The prevalence of high hypertriglyceridemia was 2.1% (1749) and of very high hypertriglyceridemia – 0.2% (173). 0.1% (108) of the subjects had mixed dyslipidemia.

In the group of people with severe dyslipidemia the prevalence of high LDL-C was 25.1% (2827), possible FH – 3.5% (389) and the frequency of definite FH was 0.6% (65) of the subjects. The prevalence of high hypertriglyceridemia was 15.5% (1749) and of very high hypertriglyceridemia– 1.5% (173). 1.0% (108) of the subjects had mixed dyslipidemia.

## Discussion

It is very important to detect and treat severe dyslipidemia as early as possible. The prevalence of the most common type of primary dyslipidemias - FH – in heterozygotes is about 1 in 200–250 of individuals of general population [7–9] and homozygotes occur with a frequency of approximately 1 per 160.000–300.000 in European populations [7, 10]. In this study the prevalence of familial hypercholesterolemia was 0.1% and possible familial hypercholesterolemia was 0.5%. The second cause of primary severe dyslipidemias - mixed dyslipidemia - is prevalent in approximately 0.5–2% of general population [11], while in this Lithuanian middle-aged primary prevention population the prevalence was 0.1%. When it comes to severe hypertriglyceridemia (SHTG), there is not enough information about the prevalence and co-morbidities of SHTG among adults. Christian J.B. and others examined the data from 5.680 adults and noticed that approximately 1.7% of the sample had SHTG (500 to 2.000 mg/dl) [12]. Another large ICARIA study reported the prevalence of 1 in 3000 for SHTG [13]. In this study the prevalence of high hypertriglyceridemia was 2.1% and very high hypertriglyceridemia – 0.2%. A clinical diagnosis of severe dyslipidemia is widely used, but a definitive diagnosis can be made by genetic screening, although mutations are currently only detected in 30–50% of patients with a clinical diagnosis of FH [14].

We did not found any similar studies investigating severe dyslipidemia. But there are many studies analyzing familial hypercholesterolemia, which could be compared with our study. Cardiovascular risk profile of severe dyslipidemia compared with results of other studies is quite similar. EUROASPIRE IV study [15], which was performed in coronary patients of 24 European countries, showed quite the same results in prevalence of BMI > 30 kg/m^2^, abdominal obesity, diabetes mellitus, good blood pressure control and physical activity. Other researchers, such as - Paul N. Hopkins and his group and Haw Neil et al. - found that older age, male gender and cigarette smoking were statistically significant risk factors of coronary heart disease (CHD) for patients with previously treated FH [16, 17]. The results of both studies showed approximately 2.5-fold increased odds ratio of ever smoking among patients with CHD compared to those without documented disease [16, 17]. Furthermore, the mean age of CHD onset is nearly four years earlier for men and women who had ever smoked [17]. In this study 22.7% of patients with severe dyslipidemia were smoking and it is slightly less than in the group without dyslipidemia. Similar percentage of smokers was observed in EUROASPIRE IV study, where 25% of patients with potential FH were smokers [15].

LitHiR programme showed that the prevalence of severe dyslipidemia was twice as high among women than among men. It should also be noted, that in general 59.1% of people participated in this study were women, although analyzed men were ten years younger. This could be explained by the fact, that men get sick and unfortunately die earlier. Therefore, there is necessity to start investigate even younger men, in order to prevent their morbidity and mortality. EUROASPIRE IV study identified the same tendency – potential FH was prevalent in 11.1% of women and in 7.5% of men surveyed [15]. The higher prevalence of potential FH for women compared to men may reflect the later onset of CHD in women in general but also for those with FH [18]. It is also important to notice, that women with severe dyslipidemia were smoking nearly four times more than men in Lithuania. What is more, women with severe dyslipidemia in comparison with men had arterial hypertension more often, they were more obese and less physically active. These findings support the theory, that atherosclerosis is a *polyetiological* chronic disease, when all factors act synergistically. High rate of smoking and higher prevalence of other risk factors found among women participating in LitHiR programme could explain the fact, that in 2015 cardiovascular disease was the leading cause of death in 65.1% of Lithuanian women, while for men this figure was 47.5% [19].

People with severe dyslipidemia are at increased risk for cardiac events, but early diagnosis, lifestyle modification and proper therapeutic intervention, which are especially needed, could help them to have a quality life.

## Conclusion

High prevalence of dyslipidemia remains a major problem in Lithuania, as 9 out of 10 people have dyslipidemia; 1 out of 10 - severe dyslipidemia. Severe dyslipidemia is associated with higher frequency of other cardiovascular risk factors. The study results show, that it is very important to focus more on earlier diagnostic, cascade screening strategy and better treatment control of patients with severe dyslipidemia to reduce their CVD risk.

## Abbreviations

BMI: body mass index
CI: confidence interval
CVD: cardiovascular disease
DM: diabetes mellitus
EUROASPIRE: European Action on Secondary Prevention through Intervention to Reduce Events
HDL-C: high density lipoprotein cholesterol
LDL-C: low density lipoprotein cholesterol
LitHiR programme: Lithuanian High Cardiovascular Risk Primary Prevention Programme
MS: metabolic syndrome
SCORE: Systematic Coronary Risk Evaluation
SD: standard deviations
SHTG: severe hypertriglyceridemia
TC: total cholesterol
